# Association of Socioeconomic Status With Long-Term Outcome in Survivors After Out-of-Hospital Cardiac Arrest: Nationwide Population-Based Longitudinal Study

**DOI:** 10.2196/47156

**Published:** 2023-07-11

**Authors:** Kyung Hun Yoo, Yongil Cho, Jaehoon Oh, Juncheol Lee, Byuk Sung Ko, Hyunggoo Kang, Tae Ho Lim, Sang Hwan Lee

**Affiliations:** 1 Department of Emergency Medicine Hanyang University Hospital Seoul Republic of Korea; 2 Department of Emergency Medicine College of Medicine Hanyang University Seoul Republic of Korea

**Keywords:** out-of-hospital cardiac arrest, OHCA, socioeconomic status, SES, long-term outcome, survivor, public health, cardiac arrest, socioeconomic disparities, hospital discharge, clinical outcomes

## Abstract

**Background:**

Out-of-hospital cardiac arrest (OHCA) is a major public health problem and a leading cause of death worldwide. Previous studies have focused on improving the survival of people who have had OHCA by analyzing short-term survival outcomes, such as the return of spontaneous circulation, 30-day survival, and survival to discharge. Research has been conducted on prehospital prognostic factors to improve the survival of patients with OHCA, among which the association between socioeconomic status (SES) and survival has been reported. SES could affect bystander cardiopulmonary resuscitation rates and whether OHCA is witnessed, and low cardiopulmonary resuscitation education rates are associated with low SES. It has been reported that areas with high SES have shorter hospital transfer times and more public defibrillators per person. Previous studies have shown the impact of SES disparities on the short-term survival of patients with OHCA. However, understanding the impact of SES on the long-term prognosis of OHCA survivors remains limited. As long-term outcomes are more indicative of a patient’s ongoing health care needs and the burden on public health than short-term outcomes, understanding the long-term prognosis of OHCA survivors is important.

**Objective:**

This study aimed to identify whether SES influenced the long-term outcomes of OHCA.

**Methods:**

Using health claims data obtained from the National Health Insurance (NHI) service in Korea, we included OHCA survivors who were hospitalized between January 2005 and December 2015. The patients were divided into 2 groups: NHI and Medical Aid (MA) groups, with the MA group defined as having a low SES. Cumulative mortality was estimated using the Kaplan-Meier method, and a Cox proportional hazards model was used to evaluate the impact of SES on long-term mortality. A subgroup analysis was performed based on whether cardiac procedures were performed.

**Results:**

We followed 4873 OHCA survivors for up to 14 years (median of 3.3 years). The Kaplan-Meier survival curve showed that the MA group had a significantly decreased long-term survival rate compared to the NHI group. With an adjusted hazard ratio (aHR) of 1.52 (95% CI 1.35-1.72), low SES was associated with increased long-term mortality. The overall mortality rate of the patients who underwent cardiac procedures in the MA group was significantly higher than that of the NHI group (aHR 1.72, 95% CI 1.05-2.82). The overall mortality rate of patients without cardiac procedures was also increased in the MA group compared to the NHI group (aHR 1.39, 95% CI 1.23-1.58).

**Conclusions:**

OHCA survivors with low SES had an increased risk of poor long-term outcomes compared with those with higher SES. OHCA survivors with low SES who have undergone cardiac procedures need considerable care for long-term survival.

## Introduction

Out-of-hospital cardiac arrest (OHCA) is a leading cause of death worldwide [1,2]. With an annual incidence among adults of 55 per 100,000 people worldwide, OHCA is a major global health concern [3,4]. Despite efforts to resuscitate people who have had cardiac arrest in communities and medical facilities, the poor prognosis for OHCA survival imposes an enormous burden on public health [2,5]. Even after being discharged from the hospital, cardiac arrest survivors face significant morbidity and mortality [6].

To achieve the best possible outcomes for adults who have had OHCA, a multifaceted approach is required. This includes early detection of cardiac arrest, activation of an emergency response, prompt administration of high-quality cardiopulmonary resuscitation (CPR), early defibrillation for shockable rhythms, and post–return of spontaneous circulation (ROSC) care [7]. However, disparities in geography, ethnicity, and socioeconomic status (SES) may impact the provision of these processes [8]. The racial and socioeconomic composition of neighborhoods can affect bystander CPR rates, and lower CPR education rates may be associated with low SES [9-11]. Patients with OHCA residing in areas with a high SES have shorter hospital transfer times and higher availability of public access defibrillators per person [12,13]. Previous studies have shown the potential impact of SES disparities on the short-term survival of patients with OHCA. A recent study reported that SES may also influence the long-term outcomes of OHCA survivors, emphasizing the need for further investigation [14]. In addition, the Institute of Medicine in the United States called for more investigation into socioeconomic differences in the outcomes of patients with OHCA [15]. However, most studies have only evaluated clinical outcomes during a short-term follow-up, such as survival to hospital discharge. Therefore, understanding the impact of SES on the long-term prognosis of OHCA survivors remains limited. Moreover, although SES is a strong predictor of cardiovascular disease prognosis, the association between SES and long-term cardiac-associated outcomes of OHCA survivors has not been investigated [16,17].

Since 1990, the Utstein Resuscitation Registry, an international standard guideline for data collection and analysis aimed at improving the outcomes of patients with OHCA, has primarily focused on short-term outcome measures: ROSC, 30-day survival, and survival to discharge [18]. However, long-term outcomes are more indicative of a patient’s ongoing health care needs and the burden on public health than short-term outcomes. With the emphasis placed on long-term prognosis, investigations are being conducted among OHCA survivors who have survived 30 days or more after OHCA, following the completion of their prehospital experience [19-21].

Therefore, this nationwide study aimed to explore the potential effect of SES on long-term outcomes among patients who survived 30 days or more after OHCA. In addition, the association between SES and long-term outcomes among OHCA survivors with and without cardiac procedures was evaluated.

## Methods

### Data Sources and Setting

We conducted a population-based study using health claims data obtained from the National Health Insurance (NHI) service. The NHI service database includes inpatient and outpatient medical information, such as patient demographics, diagnoses, procedures, drug prescriptions, date of death, and type of patient insurance (ie, NHI and Medical Aid [MA]). The diagnoses were classified based on International Classification of Diseases, 10th Revision (ICD-10) codes. All patients who visit the emergency department (ED) in South Korea for emergency symptoms, such as cardiac arrest, are charged an ED management fee. The NHI service covers a portion of the ED management fee for patients with health insurance and pays the entire fee for those with MA.

### Study Design and Population

This was a retrospective longitudinal cohort study to analyze long-term mortality. Patients who had a primary diagnosis of cardiac arrest (ICD-10 code I46.x) and were hospitalized on the day of the initial diagnosis between January 2005 and December 2015 were included. To exclude patients with in-hospital cardiac arrest, those without a primary diagnosis code for cardiac arrest or a code for ED management fees were excluded. To validate the definition of OHCA, we investigated the medical records of 252 patients who visited a tertiary hospital and had a primary diagnosis code for cardiac arrest (I46.x) and a code for ED management fees. This OHCA definition had a 92.1% (232/252) positive predictive value [22]. This study included only adult patients aged 18 years or older. Patients with OHCA who survived for 30 days or more were defined as cardiac arrest survivors, and those who survived for less than 30 days were excluded. In addition, we excluded patients with cardiac arrest resulting from nonmedical causes or trauma (ICD-10 S and T codes).

Due to the absence of medical records in NHI service data, we were unable to determine whether the cause of cardiac arrest was cardiac in origin. However, OHCA survivors who had undergone cardiac procedures could be assumed to have cardiac arrest due to a cardiac origin. Therefore, we examined whether OHCA survivors had undergone cardiac procedures such as percutaneous coronary intervention (PCI), pacemaker placement, implantable cardioverter defibrillator placement, cardiac resynchronization therapy with pacemakers, cardiac resynchronization therapy with defibrillators, and coronary artery bypass grafting during hospitalization. According to this definition, patients with OHCA were divided into cardiac procedure and no cardiac procedure groups.

### Health Insurance Systems in South Korea

In South Korea, the NHI system was introduced in 1977, and by July 1989, the entire population was covered [23]. In the past, NHI in South Korea had multiple insurance societies that covered employees and self-employed individuals separately. In 2000, however, all insurance societies were merged into a single-payer system. The NHI program covers almost 98% of the total population, which in 2014 numbered approximately 50 million people [24]. This system has been maintained until the present. The NHI service is divided into the NHI program and the MA program. The MA program is a public assistance program for low-income people who are recipients of the National Basic Livelihood Security System as part of social welfare programs, which is comparable to the Medicaid program in the United States [25]. Beneficiaries of the MA program are divided into 2 categories, types 1 and 2, based on their inability to work (including those younger than 18 years or older than 65 years and those who are disabled) and their ability to work, respectively [26]. The NHI program is divided into employed and self-employed insured groups. The insurance premium for employed and insured individuals is determined according to income and is paid by the employer. The insurance premium for self-employed individuals is based on household income, property, income, vehicles owned, age, and sex [27]. In 2020, the MA program covered 2.9% of the population, while the NHI program covered 97.1% [28]. Since the NHI service conducts compulsory collection from insured individuals in accordance with the law, the citizens of South Korea are obligated to pay insurance premiums [29].

The NHI service data include patient demographics, general specifications (eg, department, date of visit, and state), in-hospital treatment (eg, medical expenses, prescription fees, examination fees, procedure codes, and operation codes), out-of-hospital prescriptions, diagnoses, death records, and socioeconomic variables such as income decile [27,28]. Cosmetic surgery and unproven therapies are not covered by insurance [29]. The greatest value of these data is that they encompass practically the entire population, making them the closest to real-world data, which are referred to as big data [28]. These government-run national health care claims data are available to researchers for public research purposes [30]. They enable researchers to investigate all prescriptions, procedures, and operations performed by domestic medical institutions [31,32].

### SES Indicators

Individual-level indicators of SES are generally defined by income, education, and occupation [33]. The proportion of individual-level indicators differs significantly between MA and NHI beneficiaries: ordinary income level (low income: 95% vs 34%), educational level (≤elementary: 61% vs 40%), and proportion of economic activity (no economic status: 82% vs 57%) [34]. A recent study used the measurement of individual-level SES by Medicaid eligibility, which is comparable to the MA program, as a reliable indicator [35]. In addition, several previous studies used insurance type as an individual-level SES indicator [36,37]. Comparing the insurance types across NHI and MA groups can provide insights into the most vulnerable populations. Correspondingly, we categorized the patients into 2 groups: NHI and MA groups, with the MA group defined as having a low SES.

### Outcomes

The follow-up data obtained for up to 14 years (until 2018) were analyzed, and the primary outcome was long-term cumulative mortality according to SES. The subgroups were divided into OHCA with a cardiac procedure and OHCA without cardiac procedure, and the long-term cumulative mortality for each group was analyzed. Follow-up was initiated on the index date and continued until the date of death or December 31, 2018 (the last day of the study), whichever occurred first. In addition, 1-year, 3-year, 5-year, 10-year, and overall mortality were analyzed for each group. In addition, we performed an analysis by dividing the NHI group into 4 quartiles based on the insurance premium level.

### Ethics Approval

This study was approved by the Institutional Review Board of Hanyang University Hospital (HYUH-2023-01-021) and the Health Insurance Review and Assessment Service (NHIS-2023-1-122). The need for written informed consent was waived because data analyses were performed retrospectively using anonymized data derived from the NHI service. The study was conducted in accordance with the Helsinki Declaration.

### Statistical Analysis

Continuous variables are presented as the mean and SD for variables with a normal distribution and median and IQR for variables with a nonnormal distribution. Normality was evaluated using the Shapiro-Wilk test. Categorical variables are presented as frequencies and percentages. The categorical variables of 2 groups were compared using the Fisher exact test, and continuous variables were compared using the 2-tailed *t* test or Mann-Whitney *U* test as appropriate. Cumulative mortality was estimated by the Kaplan-Meier method. A Cox proportional hazards model was used to identify the impact of SES on long-term mortality, and the results are presented as hazard ratios and 95% CIs. The covariables included age, sex, and Charlson Comorbidity Index (CCI) score. The CCI score was calculated at the time of diagnosis within 1 year before the index date using the Quan algorithm [38]. By correcting the covariates, the adjusted hazard ratio (aHR) was obtained. Statistical significance was determined using 2-sided tests with a *P* value <.05. All statistical analyses were conducted using R software (version 4.2.0; R Foundation for Statistical Computing) and SAS software (version 9.4; SAS Institute Inc).

## Results

### Baseline Characteristics of the Study Population

**Figure 1:**
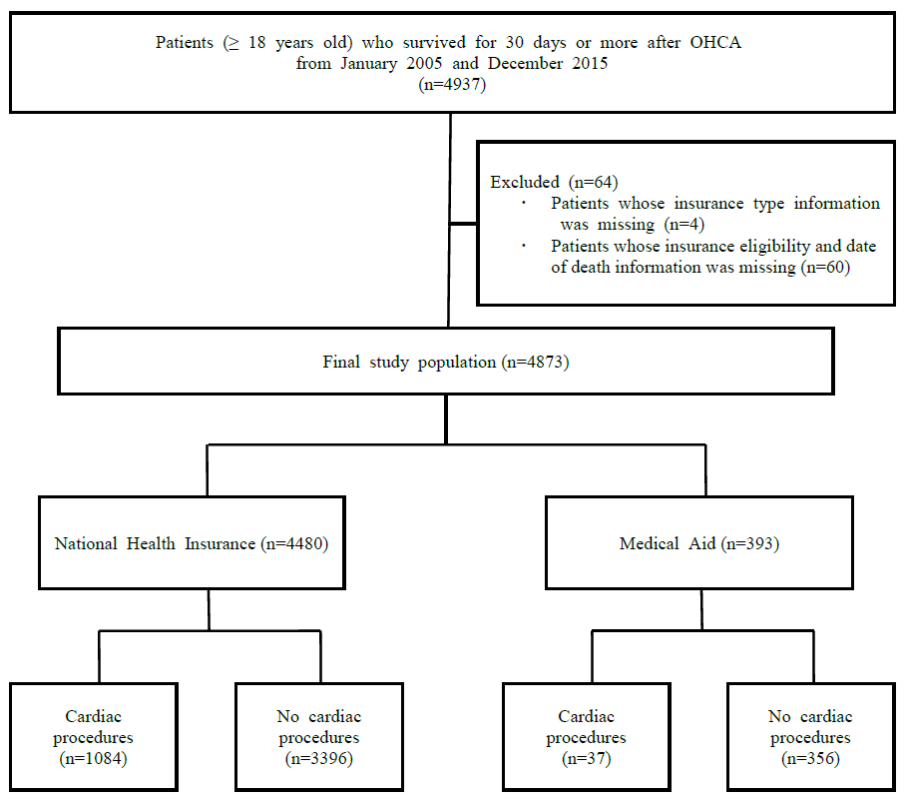
Flow diagram of the study population selection process. OHCA: out-of-hospital cardiac arrest; NHI: National Health Insurance; MA: Medical Aid.

We enrolled 4937 patients with OHCA aged 18 years or older who had survived for 30 days or more. Finally, a total of 4873 patients were selected as the study population after excluding 4 patients whose insurance type information was missing and 60 patients whose insurance eligibility and date of death information was missing (NHI: 57 of 4537 patients, MA: 3 of 396 patients). The median follow-up duration was 1214 (IQR 140-2112) days, and the maximum follow-up duration was 5059 days. The number of patients with OHCA who underwent cardiac procedures during hospitalization was 1121 (23%) (Figure 1). The cardiac procedure group’s median follow-up duration was 1768 (IQR 1.261-2.458) days, and the maximum follow-up duration was 4935 days. The no cardiac procedure group’s median follow-up duration was 716.5 (IQR 96.0-1926.8) days, and the maximum follow-up duration was 5059 days. The baseline characteristics of the study population are summarized in Tables 1 and 2.

**Table 1:** Baseline characteristics of the study population.

Characteristics			NHI^a^ group (n=4480)		MA^b^ group (n=393)
Age (years), median (IQR)			59 (48-70)		63 (52-74)
**Age (years), n (%)**					
	18-39	529 (11.8)		25 (6)	
	40-49	701 (15.6)		58 (15)	
	50-59	1130 (25.2)		89 (23)	
	60-69	951 (21.2)		83 (21)	
	70-79	802 (17.9)		84 (21)	
	≥80	367 (8.2)		54 (14)	
**Sex, n (%)**					
	Male	3190 (71.2)		211 (53.7)	
	Female	1290 (28.8)		182 (46.3)	
**CCI^c^ score, n (%)**					
	0	753 (16.8)		37 (9)	
	1	1016 (22.7)		49 (12)	
	2	913 (20.4)		70 (18)	
	3	710 (15.8)		65 (16)	
	≥4	1088 (24.3)		172 (43)	

^a^NHI: National Health Insurance.

^b^MA: Medical Aid.

^c^CCI: Charlson Comorbidity Index.

**Table 2:** Baseline characteristics of the subgroup populations.

Characteristics			Cardiac procedures (N=1129)					No cardiac procedures (N=3804)		
			NHI^a^ group (n=1084)		MA^b^ group (n=37)		NHI^a^ group (n=3396)			MA^b^ group (n=356)
Age (years), median (IQR)			54 (45-64)		60 (50-72)		60 (50-72)			63 (52-74)
**Age (years), n (%)**										
	18-39	173 (16)		5 (14)		356 (10.5)			20 (5.6)	
	40-49	215 (19.8)		4 (11)		486 (14.3)			54 (15.2)	
	50-59	320 (29.5)		9 (24)		810 (23.9)			80 (22.5)	
	60-69	230 (21.2)		9 (24)		721 (21.2)			74 (20.8)	
	70-79	124 (11.4)		5 (14)		678 (20)			79 (22.2)	
	≥80	22 (2)		5 (14)		345 (10.2)			49 (13.8)	
**Sex, n (%)**										
	Male	893 (82.4)		19 (51)		2297 (67.6)			192 (53.9)	
	Female	191 (17.6)		18 (49)		1099 (32.4)			164 (46.1)	
**CCI^c^ score, n (%)**										
	0	198 (18.3)		2 (5)		555 (16.3)			35 (9.8)	
	1	293 (27)		5 (14)		723 (21.3)			44 (12.4)	
	2	230 (21.2)		7 (19)		683 (20.1)			63 (17.7)	
	3	175 (16.1)		4 (11)		535 (15.8)			61 (17.1)	
	≥4	188 (17.3)		19 (51)		900 (26.5)			153 (43)	

^a^NHI: National Health Insurance.

^b^MA: Medical Aid.

^c^CCI: Charlson Comorbidity Index.

### Clinical Outcomes

**Figure 2:**
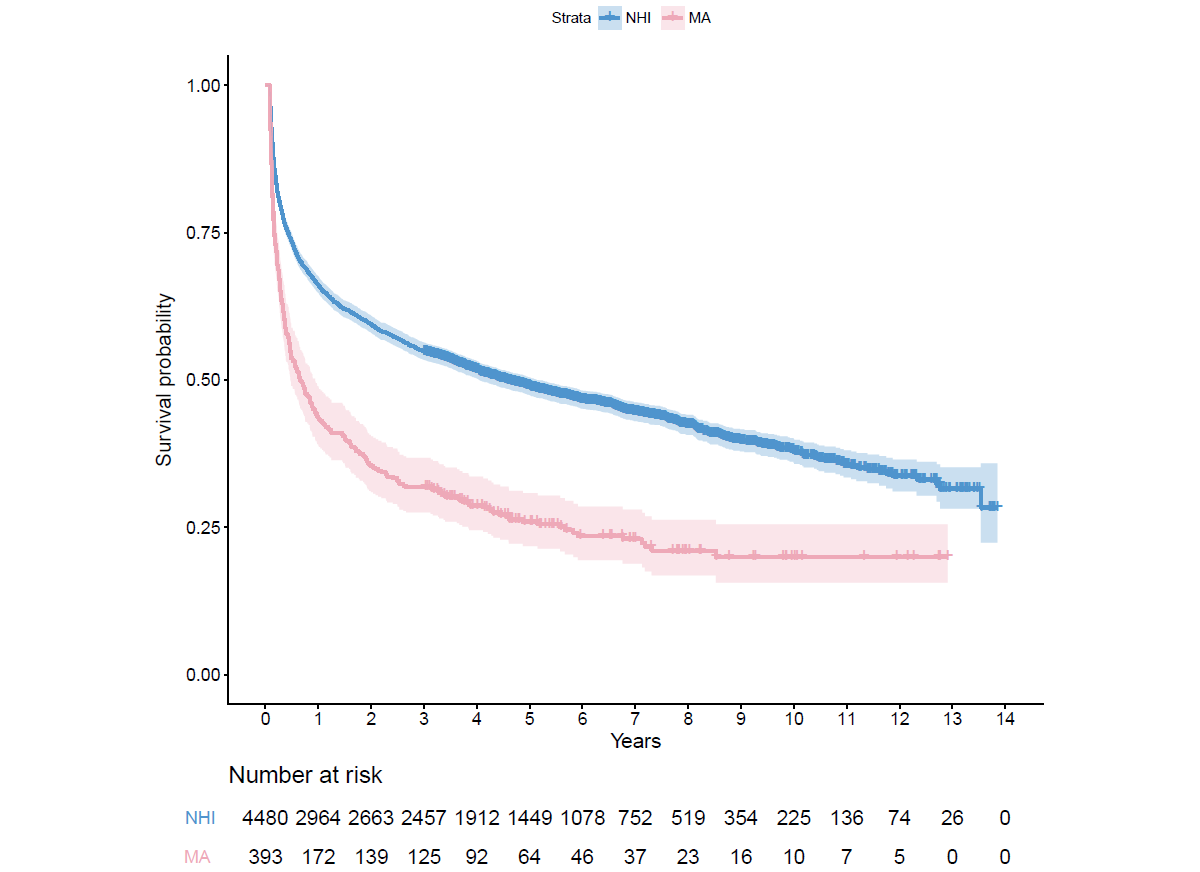
Kaplan-Meier survival curves for long-term mortality of out-of-hospital cardiac arrest survivors according to the type of patient insurance. NHI: National Health Insurance; MA: Medical Aid.

The Kaplan-Meier curve showed that the MA group had significantly decreased long-term survival compared with the NHI group (Figure 2). The results of Cox regression analysis (Table 3, all patients) showed that a low SES was associated with increased long-term mortality (aHR 1.52, 95% CI 1.35-1.72; *P*<.001).

In subgroups (Table 3, cardiac procedures and no cardiac procedures), Cox regression analysis showed that a low SES was associated with increased long-term mortality in both the cardiac procedure (aHR 1.72, 95% CI 1.05-2.82; *P*=.03) and no cardiac procedure groups (aHR 1.39, 95% CI 1.23-1.58; *P*<.001). The Kaplan-Meier curves for patients with OHCA with cardiac procedures and without cardiac procedures are presented in Multimedia Appendices 1 and 2.

**Figure 3:**
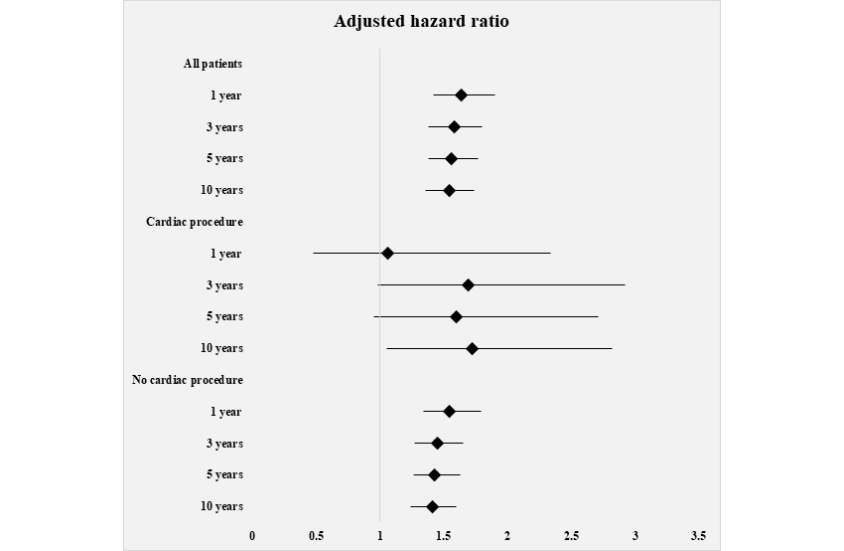
Forest plot based on the 1-year, 3-year, 5-year, and 10-year mortality results of the Medical Aid group compared with the National Health Insurance group. Adjusted hazard ratios and 95% CIs were analyzed using a Cox proportional hazards model.

The 1-year, 3-year, 5-year, and 10-year mortality rates in the MA group compared with the NHI group are presented in Table 4. The 1-year, 3-year, and 5-year mortality rates of the OHCA with cardiac procedure group were not statistically significant. However, the aHR of 10-year mortality rate in the MA group was significantly higher than that in the NHI group (aHR 1.72, 95% CI 1.05-2.82). Among all patients and in the no cardiac procedure group, the aHR of 1-year mortality rate was the highest (aHR 1.64, 95% CI 1.42-1.90; aHR 1.54, 95% CI 1.34-1.79). The forest plot based on the results of the 1-year, 3-year, 5-year, and 10-year mortality rates of the MA group compared with those of the NHI group is illustrated in Figure 3.

The baseline characteristics of the study population, divided into quartiles based on the level of insurance premiums for NHI beneficiaries, are summarized in Multimedia Appendices 3-5. Cox regression analysis revealed that the lowest quartile group and the MA group had significantly higher mortality rates compared to the highest quartile group (aHR 1.12, 95% CI 1-1.26; aHR 1.60, 95% CI 1.40-1.83, respectively). For the other quartile groups, no statistically significant differences were observed. In particular, patients who underwent cardiac procedures showed a significantly higher risk of long-term mortality (lowest quartile of NHI: aHR 1.54, 95% CI 1.08-2.18; MA: aHR 2.07, 95% CI 1.23-3.51). The MA group showed higher long-term mortality rates regardless of whether the patients had undergone cardiac procedures or not (Multimedia Appendix 6).

**Table 3:** Cox regression analysis to identify the impact of low socioeconomic status on long-term mortality among survivors of out-of-hospital cardiac arrest.

	Crude HR^a^ (95% CI)	*P* value	Adjusted HR^a^ (95% CI	*P* value
All patients	1.87 (1.65-2.11)	<.001	1.52 (1.35-1.72)	<.001
Cardiac procedures	2.67 (1.65-4.32)	<.001	1.72 (1.05-2.82)	.03
No cardiac procedures	1.57 (1.38-1.78)	<.001	1.39 (1.23-1.58)	<.001

^a^HR: hazard ratio.

**Table 4:** Cox regression analysis to identify the impact of low socioeconomic status on 1-year, 3-year, 5-year, and 10-year mortality among survivors after out-of-hospital cardiac arrest.

		Crude HR^a^ (95% CI)		Adjusted HR^a^ (95% CI
**All patients**				
	1-year mortality	1.97 (1.71-2.27)	1.64 (1.42-1.90)	
	3-year mortality	1.92 (1.69-2.18)	1.58 (1.38-1.80)	
	5-year mortality	1.91 (1.69-2.16)	1.56 (1.38-1.77)	
	10-year mortality	1.88 (1.67-2.12)	1.54 (1.36-1.74)	
**Cardiac procedures**				
	1-year mortality	1.91 (0.89-4.09)	1.06 (0.48-2.34)	
	3-year mortality	2.82 (1.66-4.78)	1.69 (0.98-2.92)	
	5-year mortality	2.57 (1.55-4.28)	1.60 (0.95-2.71)	
	10-year mortality	2.67 (1.65-4.32)	1.72 (1.05-2.82)	
**No cardiac procedures**				
	1-year mortality	1.70 (1.47-1.96)	1.54 (1.34-1.79)	
	3-year mortality	1.61 (1.41-1.84)	1.45 (1.27-1.65)	
	5-year mortality	1.61 (1.42-1.83)	1.43 (1.26-1.63)	
	10-year mortality	1.58 (1.39-1.79)	1.41 (1.24-1.60)	

^a^HR: hazard ratio.

## Discussion

### Principal Findings

This population-based study analyzed the association between long-term mortality and SES among survivors after OHCA by following these patients for up to 14 years. The long-term mortality rate of patients with a low SES was approximately 52% greater than that of patients with a higher SES. The 10-year mortality rate of patients who had undergone a cardiac procedure among OHCA survivors with a low SES was approximately >72% than that of OHCA survivors with a higher SES. With the exception of OHCA survivors in the cardiac procedure group, the aHR of 1-year mortality was highest for patients with a low SES compared to patients with a higher SES.

The majority of studies on OHCA have focused on short-term clinical outcomes, such as the ROSC, 30-day survival, and survival to discharge; however, the long-term clinical outcomes beyond 1 year are poorly understood [21]. In addition to the interest in short-term clinical outcomes, the long-term clinical outcomes and recovery of OHCA survivors have drawn attention [39]. The recovery from cardiac arrest continues long after the patient is discharged from the hospital [40,41]. As the importance of long-term prognosis has become apparent, “recovery” has been added as a new link in the chain of survival in the 2020 American Heart Association guidelines [7].

To the best of our knowledge, this is the first study to investigate the association between SES and the long-term prognoses (greater than 10 years) of OHCA survivors using data from the NHI service. In the past, the focus was on the survival of patients with OHCA, but now both the European Resuscitation Council guidelines and the American Heart Association guidelines also emphasize the importance of survivors’ recovery and rehabilitation [42,43]. From a research standpoint, this study can provide evidence for establishing a long-term strategy for OHCA survivors with a low SES.

In public health research, SES is typically measured using individual-level or area-level indicators depending on the study objectives [44,45]. Area-level SES indicators are used when a geographical area’s SES is the object of analysis rather than an individual’s SES. Area-level indicators are also often used as proxies for missing individual-level indicators in public health research, in which case they pose a risk of ecological fallacy when drawing conclusions about individuals based on area-level measurements [10,46,47]. When area-level indicators are used as proxies for individual-level SES, the SES-mortality association could be underestimated [46,48]. Long-term cumulative mortality according to SES among OHCA survivors was the primary outcome of our study. Moreover, to minimize the influence of prehospital factors associated with SES that may have area-level characteristics, such as witnessed arrest, bystander CPR, defibrillators, and the EMS system, we included patients who survived 30 days or more after OHCA. Therefore, it was considered appropriate in this study to measure SES as an individual-level indicator.

Recent studies have noted the lack of research measuring SES at the individual level [10,46,49]. Income, education, and occupation are the most traditional individual-level indicators of SES and have proven to be highly useful in describing and evaluating health disparities [45,50]. Measuring SES at the individual level usually reveals greater disparities in health outcomes and may increase awareness of the most vulnerable individuals [48,49]. However, when considered in isolation, these disparities only provide a limited view of SES inequalities in health [45]. In our additional analysis, we divided the NHI data by insurance premium level as a measure of income. There were statistically significant differences between patients in the lowest and highest quartiles who had undergone cardiac procedures. However, in the MA group, in which all 3 indicators of SES were vulnerable, significant differences were observed regardless of whether patients underwent cardiac procedures. There were significant differences between NHI beneficiaries and MA beneficiaries in terms of income, education, and economic activity. Thus, one of the strengths of our study is that it presented results of the comparison of SES as an individual-level measurement. We also provided the opportunity to compare results on a national scale by using nationwide population data.

It has been reported that a low SES is associated with poor long-term outcomes in a variety of diseases, such as cardiovascular disease, stroke, and surgery [51-53]. However, the association between long-term prognosis and SES among OHCA survivors remains unclear. Recently, Møller et al [19] reported that patients with a high income had a higher survival rate after OHCA than patients with a low income. The proportion of patients who survived 1 year after OHCA was 96.4% in the high-income group and 84.2% in the low-income group. Of a total of 1785 patients who survived 5 years after OHCA, the survival rate was 87.6% in the high-income group and 64.1% in the low-income group [19]. Our study showed that low SES had an adverse effect on the long-term outcomes of OHCA survivors, which is in line with results from a previous study. It is well known that a high SES is associated with several favorable prognostic factors for OHCA survival. However, a previous study showed that SES is also associated with the long-term outcomes of OHCA survivors, even after adjusting for witnessed arrest, bystander CPR, and initial shockable rhythm, which are well-known prehospital positive prognostic factors [19].

Although the exact etiology explaining why survivors with low SES have poor long-term outcomes after OHCA is not well understood, several hypotheses can be proposed. The usual explanation for health disparities is differences in lifestyle [54]. Individuals with a lower SES are more likely to engage in harmful alcohol use, tobacco use, unhealthy diets, and physical inactivity [55]. Medhekar et al [56] reported a similar pattern among patients with lower SES among survivors after cardiac arrest. Differences in general health due to the accumulation of these various risk factors can affect prognosis. Unfortunately, such data were not available for this study. Previous studies have shown that a low SES is associated with an increased risk of multimorbidity [57]. Likewise, our study showed that the MA group had a higher proportion of patients with multimorbidity (CCI score ≥4, 43.3% vs 24.3%). However, MA itself was independently associated with long-term mortality after adjusting for comorbidities.

It is also possible that patients with low SES receive less adequate hospital care. Post–cardiac arrest care, including critical care interventions, is another important link in the chain of survival, and post arrest interventions such as targeted temperature management and PCI are associated with improved cardiac arrest survival and neurologic recovery [42,58]. Casey and Mumma [59] showed an association between government insurance (odds ratio [OR] 0.56, 95% CI 0.51-0.61) and Medicare insurance (OR 0.44, 95% CI 0.40-0.48) and lower rates of cardiac catheterization, indicating that lower income patients may experience lower rates of PCI. Huebinger et al [60] reported that lower income, education, and occupation were all associated with lower rates of targeted temperature management and that lower income was also associated with lower rates of PCI. Although we did not investigate hospital care in this study, patients with a low SES are likely to have received fewer interventions, as the rate of MA beneficiaries among patients who underwent cardiac procedures was approximately 3.3%, and the rate of MA beneficiaries among those who did not undergo cardiac procedures was approximately 9.4%.

It is well known that individuals with a low SES have poor medication adherence [61]. Among patients with cardiovascular disease, there is a strong association between medication nonadherence and adverse clinical outcomes [62]. Therefore, adherence to medication is particularly important for OHCA survivors who have undergone cardiovascular procedures. Liang et al [63] showed that pharmacists who provided pharmaceutical services via home visits were effective in reducing medication problems among socioeconomically disadvantaged individuals. As such, efforts are needed to improve drug compliance among patients with low SES, which can also improve the long-term prognosis of OHCA survivors. Nielsen et al [64] showed that patients with low income (OR 0.2, 95% CI 0.1-0.5) and patients who lived alone (OR 0.4, 95% CI 0.2-0.9) were less likely to participate in cardiac rehabilitation (CR) programs. Meillier et al [65] suggested that inequalities in the recruitment and participation of socially vulnerable and low-educated patients in CR programs could be reduced through systematic screening and social differentiation.

Recent studies have reported the need for national financial assistance to help cardiovascular disease patients with a low SES participate in CR [66,67]. Likewise, to improve the long-term prognosis of patients with low SES among OHCA survivors, a strategy to increase rehabilitation participation and compliance with medication should be established. In addition, research on the need for national support to aid the recovery of cardiac arrest survivors is necessary. The disparity in SES will become an increasingly important public health issue [68]. The newly added link, recovery, highlights the need for the system of care to support the recovery (both short- and long-term) of cardiac arrest survivors as they return to social function [7,42]. In terms of the system of care to support the successful recovery of OHCA survivors, efforts to narrow the socioeconomic gap are important. Particularly, OHCA survivors who have undergone cardiac procedures may need public attention to improve their long-term survival for more than 10 years.

Our study has several limitations. First, the diagnosis of OHCA was defined using ICD-10 codes, and diagnostic inaccuracies could not be ruled out. Defining patients with OHCA and in-hospital cardiac arrest can be particularly imprecise. We validated the definition of OHCA, but it was based on a small sample size. Second, although this study included patients who survived 30 days or more after OHCA to minimize the influence of factors that could have affected the survival of patients with OHCA, such as initial cardiac arrest rhythm, witnessed status, bystander CPR, and duration of cardiac arrest, the potential impact could not be completely discounted. Third, because our study was based on data from a single nation, it is difficult to generalize the findings to other populations. Fourth, we only analyzed mortality as a long-term prognosis for OHCA survivors. Although mortality is an important issue in patients’ long-term outcomes, health-related quality of life (HRQOL) also has a substantial impact on long-term survival [69]. HRQOL is a multifaceted concept that comprises biological function, psychological function, and social function, which are influenced by a variety of factors [70]. Therefore, SES can influence not only the long-term mortality rate but also the HRQOL of patients. Unfortunately, it was challenging to evaluate HRQOL using NHI data. The association between SES and HRQOL among OHCA survivors needs additional investigation.

### Conclusions

This nationwide population-based study showed an association between SES and long-term outcomes among survivors after OHCA. Survivors with low SES, especially vulnerable individuals such as those eligible for MA, were at higher risk of unfavorable long-term outcomes after OHCA. Particularly, OHCA survivors with low SES who have undergone cardiac procedures need greater attention for long-term survival.

## Appendix

Multimedia Appendix 1 Kaplan-Meier survival curves for long-term mortality of out-of-hospital cardiac arrest survivors with cardiac procedures according to the type of patient insurance. NHI: National Health Insurance; MA: Medical Aid.

Multimedia Appendix 2 Kaplan-Meier survival curves for long-term mortality of out-of-hospital cardiac arrest survivors without cardiac procedures according to the type of patient insurance. NHI: National Health Insurance; MA: Medical Aid.

Multimedia Appendix 3 Baseline characteristics of the study population categorized into quartiles based on insurance premium level.

Multimedia Appendix 4 Baseline characteristics of the study population that underwent cardiac procedures categorized into quartiles based on insurance premium level.

Multimedia Appendix 5 Baseline characteristics of the study population that did not undergo cardiac procedures categorized into quartiles based on insurance premium level.

Multimedia Appendix 6 Cox regression analysis of the impact of insurance premium level on long-term mortality among survivors after out-of-hospital cardiac arrest.

## Abbreviations

aHR: adjusted hazard ratio
CCI: Charlson Comorbidity Index
CPR: cardiopulmonary resuscitation
CR: cardiac rehabilitation
ED: emergency department
HRQOL: health-related quality of life
ICD: International Classification of Diseases
MA: Medical Aid
NHI: National Health Insurance
OHCA: out-of-hospital cardiac arrest
OR: odds ratio
PCI: percutaneous coronary intervention
ROSC: return of spontaneous circulation
SES: socioeconomic status

## Data Availability

National Health Insurance Service data are third-party data that are not owned by the authors. The raw data can be accessed with permission from the National Health Insurance Service in Korea.
