# Supplementary figures and images for: Association of Socioeconomic Status With Long-Term Outcome in Survivors After Out-of-Hospital Cardiac Arrest: Nationwide Population-Based Longitudinal Study
Source: JMIR Public Health Surveill. 2023 Jul 11;9:e47156. doi: 10.2196/47156 (PMC10369165; doi:10.2196/47156)

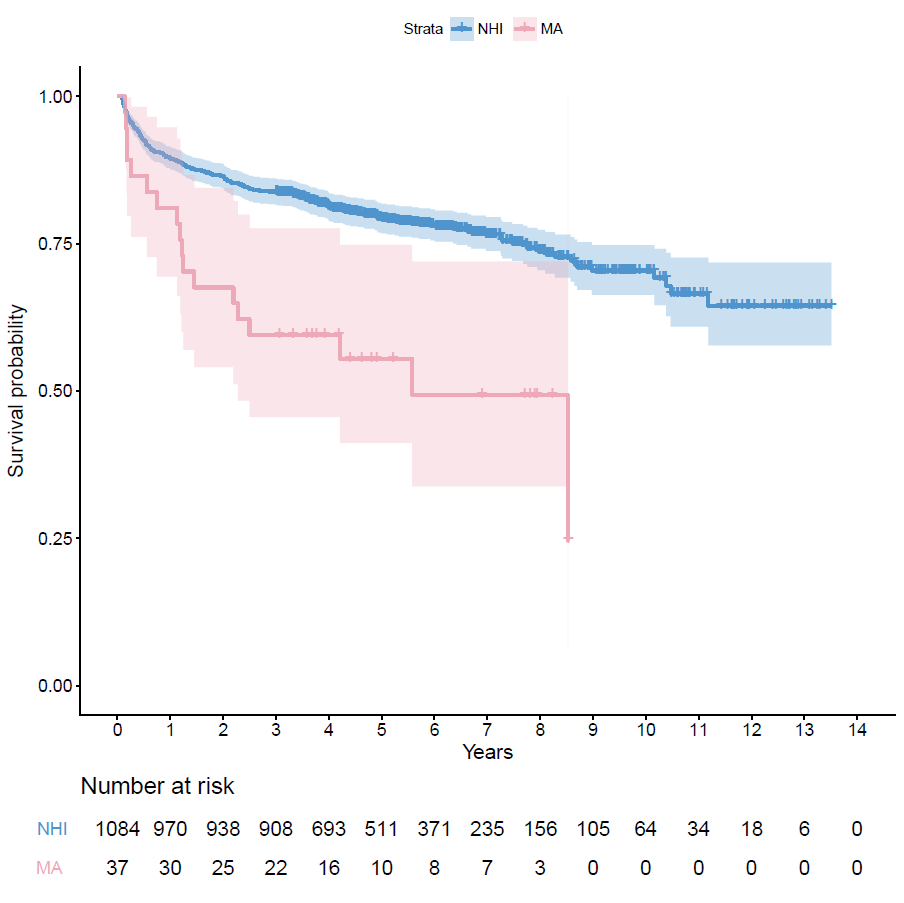

Supplement: Multimedia Appendix 1 [file publichealth_v9i1e47156_app1.png]

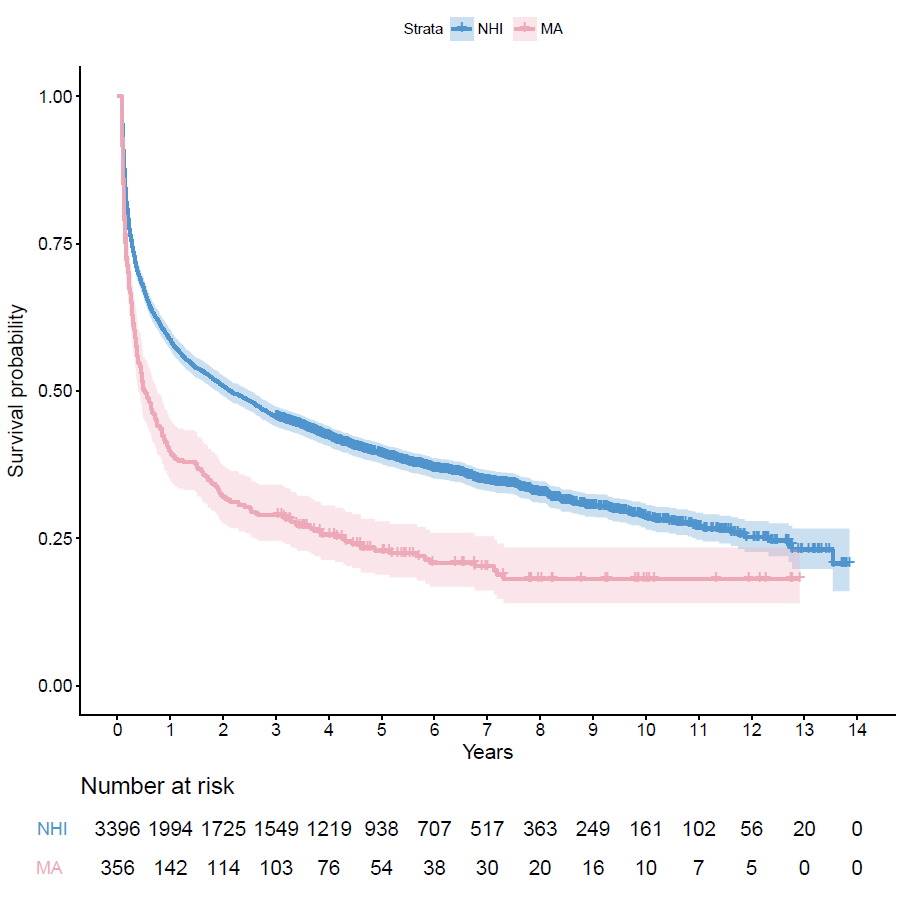

Supplement: Multimedia Appendix 2 [file publichealth_v9i1e47156_app2.png]
