# Supplementary material for: Association of Socioeconomic Status With Long-Term Outcome in Survivors After Out-of-Hospital Cardiac Arrest: Nationwide Population-Based Longitudinal Study
Source: JMIR Public Health Surveill. 2023 Jul 11;9:e47156. doi: 10.2196/47156 (PMC10369165; doi:10.2196/47156)
Supplement: Multimedia Appendix 3 [file publichealth_v9i1e47156_app3.docx]

Multimedia Appendices 3. Baseline characteristics of the study population categorized into quartiles based on insurance premium level.

|  | Q4  (N=1,556) | Q3  (N=1,067) | Q2  (N=900) | Q1  (N=874) | MA  (N=393) |
| --- | --- | --- | --- | --- | --- |
| Age, median (25^th^-75^th^ percentile) | 64 (50-70) | 58 (46-68) | 56 (47-65) | 57 (52-74) | 63 (52-74) |
| Age category (n, %) |  |  |  |  |  |
| 18-39 | 131 (8.4) | 154 (14.4) | 130 (14.4) | 106 (12.1) | 25 (6.4) |
| 40-49 | 229 (14.7) | 173 (16.2) | 156 (17.3) | 134 (15.3) | 58 (14.8) |
| 50-59 | 316 (20.3) | 263 (24.6) | 283 (31.4) | 246 (28.1) | 89 (22.6) |
| 60-69 | 287 (18.4) | 261 (24.5) | 177 (19.7) | 207 (23.7) | 83 (21.1) |
| 70-79 | 382 (24.6) | 161 (15.1) | 108 (12.0) | 134 (15.3) | 84 (21.4) |
| ≥80 | 211 (13.6) | 55 (5.2) | 46 (5.1) | 47 (5.4) | 54 (13.7) |
| Sex (n, %) |  |  |  |  |  |
| Male | 1,091 (70.1) | 761 (71.3) | 651 (72.3) | 627 (71.7) | 211 (53.7) |
| Female | 465 (29.9) | 306 (28.7) | 549 (27.7) | 247 (28.3) | 182 (46.3) |
| CCI score (n, %) |  |  |  |  |  |
| 0 | 240 (15.4) | 176 (16.5) | 155 (17.2) | 162 (18.5) | 37 (9.4) |
| 1 | 339 (21.8) | 243 (22.8) | 213 (23.7) | 204 (23.3) | 49 (12.5) |
| 2 | 332 (21.3) | 207 (19.4) | 179 (19.9) | 184 (21.1) | 70 (17.8) |
| 3 | 247 (15.9) | 173 (16.2) | 137 (15.2) | 143 (16.4) | 65 (16.5) |
| ≥4 | 398 (25.6) | 268 (25.1) | 216 (24.0) | 181 (20.7) | 172 (43.8) |
