# Supplementary material for: Association of Socioeconomic Status With Long-Term Outcome in Survivors After Out-of-Hospital Cardiac Arrest: Nationwide Population-Based Longitudinal Study
Source: JMIR Public Health Surveill. 2023 Jul 11;9:e47156. doi: 10.2196/47156 (PMC10369165; doi:10.2196/47156)
Supplement: Multimedia Appendix 4 [file publichealth_v9i1e47156_app4.docx]

Multimedia Appendices 4. Baseline characteristics of the study population that underwent cardiac procedures categorized into quartiles based on insurance premium level.

|  | Q4  (N=372) | Q3  (N=268) | Q2  (N=217) | Q1  (212) | MA  (N=37) |
| --- | --- | --- | --- | --- | --- |
| Age, median (25^th^-75^th^ percentile) | 55 (47-67) | 53 (41-62) | 52 (42-60) | 55 (47-63) | 60 (50-72) |
| Age category (n, %) |  |  |  |  |  |
| 18-39 | 44 (11.8) | 57 (21.3) | 44 (20.3) | 27 (12.7) | 5 (13.5) |
| 40-49 | 71 (19.1) | 56 (20.9) | 44 (20.3) | 42 (19.8) | 4 (10.8) |
| 50-59 | 113 (30.4) | 67 (25.0) | 71 (32.7) | 65 (30.7) | 9 (24.3) |
| 60-69 | 68 (18.3) | 63 (23.5) | 39 (18.0) | 54 (25.5) | 9 (24.3) |
| 70-79 | 59 (15.9) | 24 (9.0) | 16 (7.4) | 23 (10.8) | 5 (13.5) |
| ≥80 | 17 (4.6) | 1 (0.4) | 3 (1.4) | 1 (0.5) | 5 (13.5) |
| Sex (n, %) |  |  |  |  |  |
| Male | 314 (84.4) | 223 (83.2) | 171 (78.8) | 171 (80.7) | 19 (51.4) |
| Female | 58 (15.6) | 45 (16.8) | 46 (21.2) | 41 (19.3) | 18 (48.6) |
| CCI score (n, %) |  |  |  |  |  |
| 0 | 72 (19.4) | 43 (16.0) | 45 (20.7) | 35 (16.5) | 2 (5.4) |
| 1 | 100 (26.9) | 77 (28.7) | 46 (21.2) | 66 (31.1) | 5 (13.5) |
| 2 | 73 (19.6) | 63 (23.5) | 53 (24.4) | 37 (17.5) | 7 (18.9) |
| 3 | 57 (15.3) | 41 (15.3) | 36 (16.6) | 41 (19.3) | 4 (10.8) |
| ≥4 | 70 (18.8) | 44 (16.4) | 37 (17.1) | 33 (15.6) | 19 (51.4) |
