# Supplementary material for: Association of Socioeconomic Status With Long-Term Outcome in Survivors After Out-of-Hospital Cardiac Arrest: Nationwide Population-Based Longitudinal Study
Source: JMIR Public Health Surveill. 2023 Jul 11;9:e47156. doi: 10.2196/47156 (PMC10369165; doi:10.2196/47156)
Supplement: Multimedia Appendix 5 [file publichealth_v9i1e47156_app5.docx]

Multimedia Appendices 5. Baseline characteristics of the study population that did not undergo cardiac procedures categorized into quartiles based on insurance premium level.

|  | Q4  (N=1184) | Q3  (N=799) | Q2  (N=683) | Q1  (N=662) | MA  (N=356) |
| --- | --- | --- | --- | --- | --- |
| Age, median (25^th^-75^th^ percentile) | 67 (53-76) | 59 (48-69) | 57 (48-67) | 59 (49-69) | 63 (52-74) |
| Age category (n, %) |  |  |  |  |  |
| 18-39 | 87 (7.3) | 97 (12.1) | 86 (12.6) | 79 (11.9) | 20 (5.6) |
| 40-49 | 158 (13.3) | 117 (14.6) | 112 (16.4) | 92 (13.9) | 54 (15.2) |
| 50-59 | 203 (17.1) | 196 (24.5) | 212 (31.0) | 181 (27.3) | 80 (22.5) |
| 60-69 | 219 (18.5) | 198 (24.8) | 138 (20.2) | 153 (23.1) | 74 (20.8) |
| 70-79 | 323 (27.3) | 137 (17.1) | 92 (13.5) | 111 (16.8) | 79 (22.2) |
| ≥80 | 194 (16.4) | 54 (6.8) | 43 (6.3) | 46 (6.9) | 49 (13.8) |
| Sex (n, %) |  |  |  |  |  |
| Male | 777 (65.6) | 538 (67.3) | 480 (70.3) | 456 (68.9) | 192 (53.9) |
| Female | 407 (34.4) | 261 (32.7) | 203 (29.7) | 206 (31.1) | 164 (46.1) |
| CCI score (n, %) |  |  |  |  |  |
| 0 | 168 (14.2) | 133 (16.6) | 110 (16.1) | 127 (19.2) | 35 (9.8) |
| 1 | 239 (20.2) | 166 (20.8) | 167 (24.5) | 138 (20.8) | 44 (12.4) |
| 2 | 259 (21.9) | 144 (18.0) | 126 (18.4) | 147 (22.2) | 63 (17.7) |
| 3 | 190 (16.0) | 132 (16.5) | 101 (14.8) | 102 (15.4) | 61 (17.1) |
| ≥4 | 328 (27.7) | 224 (28.0) | 179 (26.2) | 148 (22.4) | 153 (43.0) |
