# Supplementary material for: Association of Socioeconomic Status With Long-Term Outcome in Survivors After Out-of-Hospital Cardiac Arrest: Nationwide Population-Based Longitudinal Study
Source: JMIR Public Health Surveill. 2023 Jul 11;9:e47156. doi: 10.2196/47156 (PMC10369165; doi:10.2196/47156)
Supplement: Multimedia Appendix 6 [file publichealth_v9i1e47156_app6.docx]

Multimedia Appendices 6. Cox regression analysis of the impact of insurance premium level on long-term mortality among survivors after out-of-hospital cardiac arrest.

|  | All patients | | Cardiac procedures | | No cardiac procedures | |
| --- | --- | --- | --- | --- | --- | --- |
|  | Crude HR | 95% CI | Crude HR | 95% CI | Crude HR | 95% CI |
|  |  |  |  |  |  |  |
| Q1 (Ref) |  |  |  |  |  |  |
| Q2 | 0.89 | 0.80-0.99 | 0.99 | 0.70-1.40 | 0.88 | 0.79-0.98 |
| Q3 | 0.80 | 0.72-0.90 | 0.99 | 0.69-1.43 | 0.77 | 0.68-0.87 |
| Q4 | 0.94 | 0.84-1.05 | 1.32 | 0.94-1.87 | 0.89 | 0.79-1.00 |
| MA | 1.73 | 1.51-1.97 | 2.85 | 1.71-4.77 | 1.41 | 1.23-1.62 |
|  |  |  |  |  |  |  |
|  | Adjusted HR | 95% CI | Adjusted HR | 95% CI | Adjusted HR | 95% CI |
|  |  |  |  |  |  |  |
| Q1 (Ref) |  |  |  |  |  |  |
| Q2 | 1.07 | 0.96-1.19 | 1.18 | 0.83-1.66 | 1.04 | 0.93-1.17 |
| Q3 | 0.02 | 0.91-1.15 | 1.27 | 0.88-1.84 | 0.96 | 0.85-1.09 |
| Q4 | 1.12 | 1.00-1.26 | 1.54 | 1.08-2.18 | 1.06 | 0.94-1.19 |
| MA | 1.60 | 1.40-1.83 | 2.07 | 1.23-3.51 | 1.42 | 1.24-1.63 |
